# Development and Field Evaluation of the INTER-ACT App, a Pregnancy and Interpregnancy Coaching App to Reduce Maternal Overweight and Obesity: Mixed Methods Design

**DOI:** 10.2196/16090

**Published:** 2020-02-14

**Authors:** Annick Bogaerts, Margriet Bijlholt, Lotte Mertens, Marijke Braeken, Bart Jacobs, Bert Vandenberghe, Lieveke Ameye, Roland Devlieger

**Affiliations:** 1 Department of Development and Regeneration University of Leuven Leuven Belgium; 2 Faculty of Health and Social Work, Research Unit Resilient People University Colleges Leuven-Limburg Leuven Belgium; 3 Faculty of Medicine and Health Sciences, Centre for Research and Innovation in Care University of Antwerp Antwerp Belgium; 4 Faculty of Rehabilitation Sciences Rehabilitation Research Center, Biomedical Research Institute Hasselt University Hasselt Belgium; 5 Meaningful Interactions Lab (Mintlab) University of Leuven Leuven Belgium; 6 Department of Obstetrics and Gynecology University Hospitals Leuven Leuven Belgium; 7 Department of Obstetrics, Gynecology and Fertility Gasthuiszusters Antwerpen, Campus Sint-Augustinus Wilrijk Belgium

**Keywords:** pregnancy, postpartum, coaching, lifestyle, mobile app

## Abstract

**Background:**

The interpregnancy and pregnancy periods are important windows of opportunity to prevent excessive gestational weight retention. Despite an overwhelming number of existing health apps, validated apps to support a healthy lifestyle between and during pregnancies are lacking.

**Objective:**

To describe the development and evaluation of the INTER-ACT app, which is part of an interpregnancy and pregnancy lifestyle coaching module, to prevent excessive weight gain in pregnancy and enhance optimal weight and a healthy lifestyle in the interpregnancy period.

**Methods:**

A mixed methods design was used to identify the needs of health care providers and end users, according to 15 semistructured interviews, two focus groups, and two surveys. The user interface was evaluated in a pilot study (N=9).

**Results:**

Health care providers indicated that a mobile app can enhance a healthy lifestyle in pregnant and postpartum women. Pregnant women preferred graphic displays in the app, weekly notifications, and support messages according to their own goals. Both mothers and health care providers reported increased awareness and valued the combination of the app with face-to-face coaching.

**Conclusions:**

The INTER-ACT app was valued by its end users because it was offered in combination with face-to-face contact with a caregiver.

## Introduction

An increasing number of women are obese at the start of pregnancy. Concurrently, one in three European pregnant women has excessive gestational weight gain [1]. In particular, women with a high pregestational body mass index (BMI), young women (<20 years), single women, and women belonging to ethnic minority groups are at risk [2]. Adverse outcomes associated with maternal obesity and excessive gestational weight gain include gestational hypertension, gestational diabetes mellitus, and large-for-gestational age infants [3]. Approximately half of women with excessive gestational weight gain do not return to their prepregnancy BMI before the next pregnancy. This increases prepregnancy obesity and is an important predictor for increased risks of pregnancy- and birth-related outcomes in the next pregnancy, including cesarean delivery, fetal overgrowth, and postnatal weight retention [3-6].

Face-to-face lifestyle intervention studies during pregnancy are effective to reduce gestational weight gain [7-9], but they are time-consuming with limited scalability, and no or minimal effects have been shown regarding relevant pregnancy outcomes [10-12]. Given the high impact of prepregnancy BMI, intervening early during the preconception period is essential [3]. Reaching the most vulnerable women and subsequently achieving adherence to a healthy lifestyle before becoming pregnant are of high priority [13].

The use of mobile health (mHealth) technology in the prevention, screening, and treatment of health-related issues is increasing, as is reflected by the ample offering of smartphone apps. On one hand, mHealth can offer easier access to individually-tailored support at a low cost. On the other hand, these apps are mostly not targeted at groups with specific needs, such as pregnant and postnatal (between pregnancies) women. Moreover, their effectiveness has not been tested in randomized controlled trials (RCTs) [14]. Results of the effectiveness of mHealth tools are scarce [15,16], but pioneering studies have shown promising results regarding intervention adherence, feasibility, and achieving an adequate pregnancy weight gain [17-19].

The aim of this study was therefore twofold. First, we aimed to develop an app to monitor and coach pregnant and postnatal women with focus on maternal weight, physical activity, healthy eating, and mental wellbeing. Second, we aimed to gather feedback on user experience (ie, usability, usefulness, and user acceptance). This app, called INTER-ACT, will be used in combination with four postnatal (interpregnancy) and three prenatal face-to-face coaching sessions. The ultimate aim of an RCT, in which this app is embedded, is to reduce the risk of gestational hypertension, gestational diabetes, cesarean section, and large-for-gestational-age infants in subsequent pregnancy among women who had excessive gestational weight gain in their previous pregnancy [19].

## Methods

### Overview

The INTER-ACT app targets women during the interpregnancy period, as well as pregnant women. The interpregnancy period is defined as the period between delivery and the start of a subsequent pregnancy.

**Figure 1:**
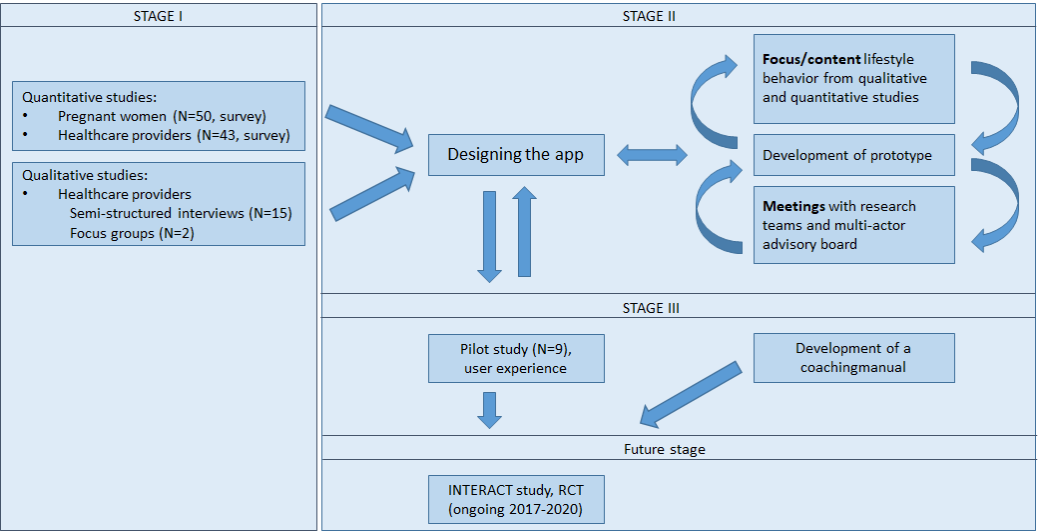
Flow chart of the three stages of app development (data collection, app development, and field evaluation). RCT: randomized controlled trial.

The app was developed in three stages (Figure 1). First, a mixed methods design was used to gain insights into experiences with and views on perinatal lifestyle coaching from the perspective of health care providers and women/end users. Second, the app was designed by user-experience researchers and developed by the Belgium Campus ITversity in South Africa. Third, the app was evaluated in a qualitative field evaluation study. The three stages are elaborated below. A subsequent stage that is beyond the scope of this study involves embedding the app in a lifestyle intervention and evaluating it with an RCT design. The content of the face-to-face coaching is described elsewhere [19].

### Stage I: Insights From Caregivers and End Users

#### Health Care Providers’ Perspectives

We conducted semistructured interviews with a purposive sample of four general practitioners, three gynecologists, five midwives, and three dieticians (Table 1), who were selected according to their previous experience with obesity care in pregnant and postnatal women. A topic list was developed to gain insight into their experiences with and views on perinatal lifestyle coaching and their attitude towards technology-supported lifestyle coaching. In addition, two focus groups with a total of 16 midwives were conducted to explore their experiences with and views on perinatal lifestyle coaching and their attitude towards technology-supported lifestyle coaching in order to support data triangulation and achieve data saturation. All interviews and focus groups were audiotaped, transcribed, and analyzed thematically using open coding. The analysis of focus groups additionally included a peer debriefing with our researchers to control the interpretation of the results. Furthermore, 43 caregivers (Table 2) attending a symposium about lifestyle coaching in pregnant women were asked to respond to two open questions about their knowledge and skills regarding perinatal lifestyle coaching and potential gaps. Written informed consent was obtained from all participants, and confidentiality and anonymity were assured. Ethical approval was obtained from University Hospital Universitair Ziekenhuis Leuven, Belgium (B300201422650).

**Table 1:** Professions of health care providers participating in interviews and focus groups (N=31).

Profession	Frequency, n (%)
Midwife in primary care	13 (42)
Midwife in secondary care	5 (16)
Midwife in primary and secondary care	3 (10)
General practitioner	4 (13)
Gynecologist	3 (10)
Dietician	3 (10)

**Table 2:** Professions of health care providers participating in answering two open questions (N=43).

Profession	Frequency, n (%)
Midwife in primary care	3 (7)
Midwife in secondary care	16 (37)
Midwife (not in direct care)	9 (21)
Dietician	3 (7)
Nurse	3 (7)
Student	6 (14)
Teacher	3 (7)

#### End Users’ Needs

We conducted a survey among 50 pregnant women between 12 and 42 weeks of pregnancy (Table 3) to explore their needs regarding technology-supported lifestyle coaching to optimize gestational weight gain. They were recruited from the waiting room before prenatal consultations in two nonuniversity hospitals. The inclusion criteria were as follows: sufficient fluency in spoken Dutch, age between 18 and 45 years, uncomplicated pregnancy between 12 and 42 weeks, and at least one prenatal consultation prior to the current consultation. The exclusion criteria were as follows: twin pregnancies, diagnosis of gestational diabetes or complications influencing physical activity or eating behavior. Ethical approval was obtained from University Hospital Universitair Ziekenhuis Leuven, Belgium (B243201628083).

**Table 3:** Characteristics of the survey participants (pregnant women) (N=50).

Characteristic		Value, n (%)	
**Gestational age (weeks)**			
	First trimester (0-14)		8 (16)
	Second trimester (15-27)		10 (20)
	Third trimester (28-40)		32 (64)
**Gravidity**			
	Nullipara		33 (66)
	Multipara		17 (34)
**BMI^a^ group**			
	Underweight (<18.5)		7 (14)
	Normal weight (18.5-24.9)		23 (46)
	Overweight (25-29.9)		12 (24)
	Obesity class I (30-34.9)		4 (8)
	Obesity class II (35-39.9)		1 (2)
	Obesity class III (≥40)		2 (4)
**Method of conception**			
	Spontaneous		44 (88)
	Assisted reproduction		6 (12)
**Age (years)**			
	18-24		5 (10)
	25-29		23 (46)
	30-34		16 (32)
	35-39		5 (10)
	40-44		1 (2)
**Education**			
	Primary education		2 (4)
	Secondary education		21 (42)
	Bachelor’s degree		15 (30)
	Master’s degree		13 (26)
**Nationality**			
	Belgian		46 (92)
	Dutch		2 (4)
	Others		2 (4)
**Marital status**			
	Married		30 (60)
	Cohabiting		19 (38)
	Single		1 (2)

^a^BMI: body mass index.

### Stage II: App Development

**Figure 2:**
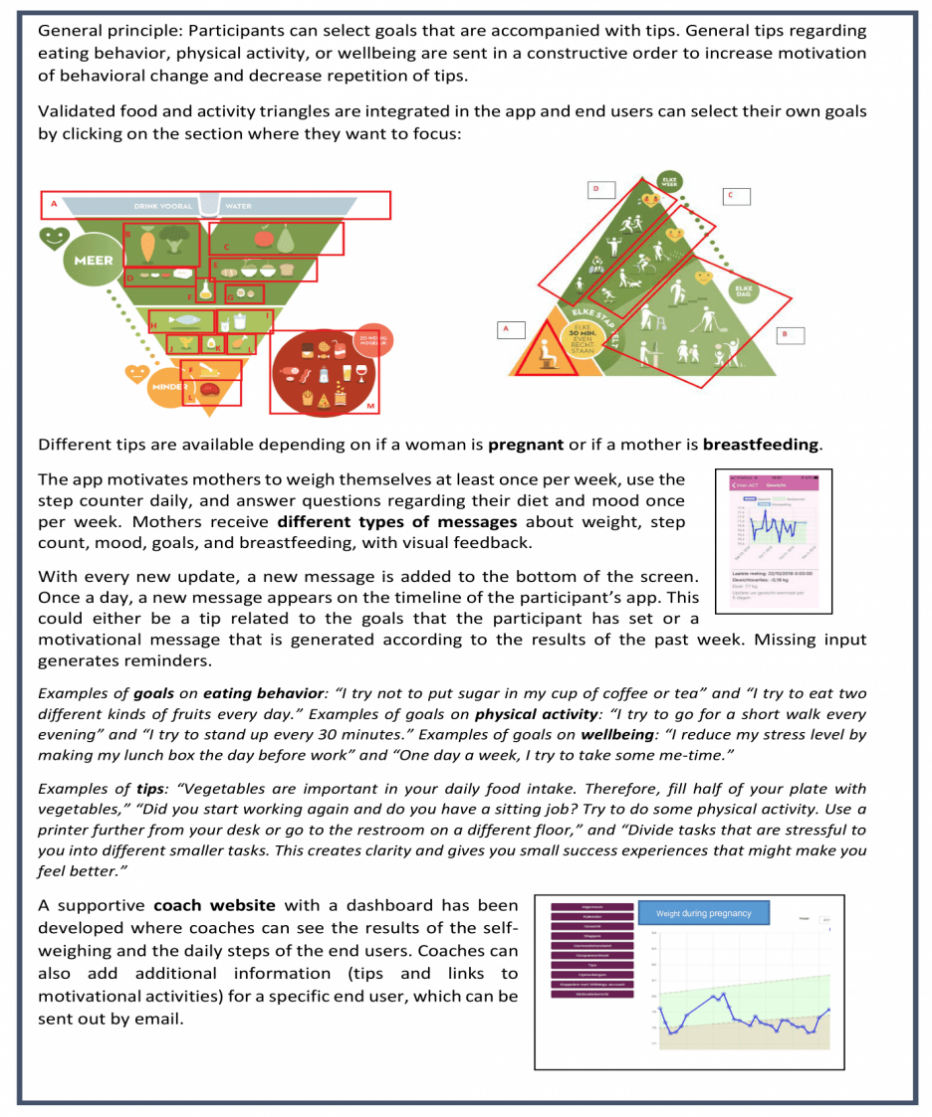
Description of the functionality of the INTER-ACT app and examples of messages.

The content of the app was based on the nutritional recommendations of the Superior Health Council of Belgium and the Institute of Medicine guidelines for gestational weight gain [20]. Additionally, guidelines from the Flemish Institute for Healthy Living and results from discussions with experts (clinicians, researchers, and policy makers) on the INTER-ACT external advisory board contributed to the content of the app. Furthermore, the principles of motivational interviewing techniques, goal setting, and positive messaging were incorporated in the app. User-experience researchers designed the INTER-ACT app (Figure 2) according to usability heuristics, state-of-the-art insights from the domain of human-computer interaction, research experiences from previous mHealth projects and technologies [21], and results from the interviews and focus groups described in the first stage.

The participants could use INTER-ACT to monitor mental wellbeing, set goals on physical activity and healthy eating, and record progress on these goals. Additionally, Bluetooth connections were made with the Withings Go activity tracker (model WAM02; Withings, Issy-les-Moulineaux, France) and Withings Body+ weighing scale (model WBS05; Withings) in order to track physical activity and weight, respectively. Tips and motivating messages to support weight management, physical activity, healthy eating, and mental wellbeing were created, and an algorithm was developed to send these messages to the participants according to their input. Custom tips could be added by the researchers and sent to specific participants.

**Figure 3:**
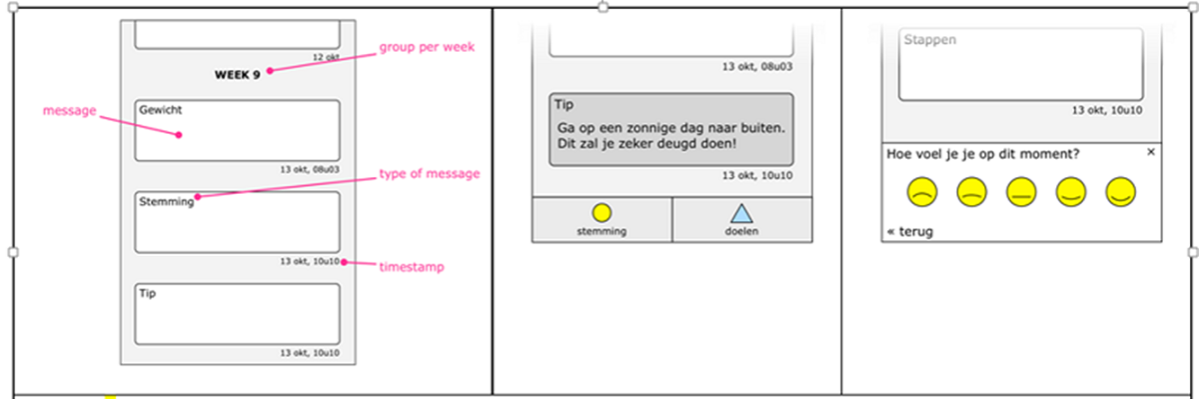
Wireframes of the INTER-ACT app: stream of messages (left), coaching message (center), and manual input of mood (right).

The user interface of the app is designed according to the principle of conversational interfaces [22]. All content in the app is structured as a conversation between the user and the system in a chronological stream of messages (eg, a new step count or weight) (Figure 3). Messages are clickable, and clicking opens a page that provides additional information regarding the clicked message (eg, a weight graph). This approach allows the combination of both automatic input (from the weighing scale and activity tracker) and manual input (from entered mood), the display of feedback on achieved goals, and the display of reminders after a period of nonuse in a dynamic way.

The first prototype of the app was tested for functionality and feasibility by two pregnant women and a multidisciplinary team involving a professor of gynecology, a professor of midwifery, a biostatistician, a psychologist, two lifestyle coaches, and a group of app developers. They provided feedback regarding the design from medical, wellbeing, and technical perspectives. An iterative process of adaptation led to the development of the INTER-ACT app, which is being used in an ongoing RCT.

To ensure privacy and data security, the data are stored in a database hosted at a secure data center in Katholieke Universiteit Leuven. The database can only be accessed by our Application Programming Interface via a Structured Query Language connection. Security between the Application Programming Interface and end users involves a username and password system to keep the approach user friendly, but in the background, this system is supported by token-based authentication to prevent password theft. For the external system, we access authentication for data transfer via the Withings OAUTH2 system (Withings).

### Stage III: Field Evaluation

The app was assessed in a qualitative field evaluation study, in which the technical functionality and user experience were explored. We recruited two pregnant and seven postnatal women (<6 months after delivery) through social media. During a home visit, the researchers installed the app, set up the Withings Go activity tracker and Withings Body+ weighing scale, and provided a short explanation of the app functions. The women used the app and devices for 3 weeks and were contacted at least once a week by telephone to address potential usability issues with the app and devices. In case of questions, the women could also contact the researchers by email and telephone. After the 3-week period, a semistructured interview was conducted during a home visit. Technical functionality issues, such as crashes, bugs, and connectivity issues with the activity tracker and weighing scale, were explored. The evaluation of user experience involved topics, such as content of knowledge- and skill-based elements; content, number, and timing of notifications; experienced accuracy of the activity tracker; and esthetics. The interviews were recorded and transcribed verbatim for analysis. The researchers’ written notes of the observations made during the home visits, user feedback of the app, and reported user experiences were analyzed through an affinity diagram using Post-It notes. These insights allowed us to improve the app for a better user experience and prepare it for a full-scale field trial. Ethical approval was obtained for all studies, and informed consent was provided by all respondents (University Hospital Universitair Ziekenhuis Leuven, Belgium; B322201730956).

## Results

### Health Care Providers’ Perspectives

Qualitative semistructured interviews and focus group discussions revealed health care provider–experienced barriers and facilitators, and perspectives on pregnancy and postpartum lifestyle coaching supported by mHealth. According to health care providers, low social background and educational levels, increased economic difficulties, ethnic minorities, different cultural or religious context, and insufficient knowledge about healthy eating were characteristics that needed attention in performing lifestyle coaching. The experienced facilitating factors were women’s motivation to change lifestyle, awareness of their own responsibility, and self-control. Some health care providers were not convinced that an app would be effective in acquiring a healthier lifestyle among obese pregnant women, and they felt that it could even induce fear and anxiety. From the open questionnaires (n=43) and interviews (n=15), the following three themes for coaching emerged: (1) in-depth communication training; (2) motivational techniques; and (3) behavioral change training, with specific attention to sensitive communication for vulnerable groups, including insights on their religious and cultural contexts.

During the focus groups with midwives, they indicated a willingness to take up the role of a coach to empower women for a healthy lifestyle, but they lacked practical knowledge and skills to support vulnerable groups. They were not sure whether an app would be helpful in lifestyle coaching. However, if combined with face-to-face coaching and not used as a tool to “monitor and control” women’s behavior, they indicated that an app could be useful. Data collected in the app could facilitate a coaching session and could result in a conversation about healthy lifestyle issues. However, midwives expressed that they prefer to restrict their administrative work and do not want to spend time on integration of additional technologies.

### End Users’ Needs

Among the 50 pregnant women who completed the survey, 30 (60%) wanted personal advice from caregivers about a healthy lifestyle. Only 8 out of 15 women (16%) indicated currently being counselled, mostly only regarding prenatal weight management (Table 4).

Additionally, 45 out of the 50 women (90%) indicated that an app would help them to maintain a healthy lifestyle. Among the 50 women, 46 (92%) were eager to monitor their calorie consumption and 28 (56%) were eager to monitor physical activity goals using an app or diary. Moreover, among the 50 women, 45 (90%) indicated that they would like to self-monitor their mental wellbeing using a Likert scale with emoticons and 39 (78%) indicated that encouraging messages might enhance their motivation. Furthermore, among the 50 women, 36 (72%) preferred the app to display and evaluate the actual weight and weight gain, including tailored feedback. All women preferred an app that could tell them what they could eat safely in pregnancy and that included food diaries, weekly shopping lists, and pictures with recommended portion sizes (Table 4).

The women indicated that the attractiveness of the app might be enhanced by the addition of features regarding fetal development, an agenda for prenatal appointments, a checklist with hospital necessities, information on health risks for the mother and child, the ability to upload pictures and ultrasounds, a contraction counter, and a kick counter. Finally, women reported that they want their partners to be involved in the use of the app.

### Field Evaluation

The qualitative user evaluation study showed a high user acceptance of the system and reported an increased consciousness regarding physical activity, eating behavior, weight management, and mental wellbeing. The activity tracker, goal setting for nutrition, and regular push notifications were especially appreciated.

Multiple users requested to increase the number of notifications and suggested to spread them during the day instead of a single evening notification. Furthermore, users preferred to configure both the kind of reminder (steps, weight, mood, and goals based on the user’s own behavior) and the timing.

Participants who had an app and device installed on their smartphones besides the INTER-ACT app made comparisons between the two apps (eg, comparisons were made regarding the accuracy of the activity tracker). Participants rarely felt that the Withings Go activity tracker was more accurate than their known devices (eg, Fitbit). There were no such remarks regarding the weighing scale. Participants reported missing certain functionalities that other health- and weight-related apps incorporate, such as sleep tracking, heart-rate monitoring, and advanced food tracking and calorie counting.

The esthetics of our study app were considered less modern or attractive when compared with today’s standard. Despite these remarks, our participants noted important value in the INTER-ACT app when combined with face-to-face coaching.

**Table 4:** Results from the survey of pregnant women (N=50).

Factor		Value, n (%)
**Preferred personal lifestyle advice**		
	Yes	30 (60)
	No	20 (40)
**Current lifestyle follow-up by a health care provider**		
	Yes	8 (16)
	No	42 (84)
**If yes, focus of current lifestyle follow-up by a health care provider**		
	Weight	4 (8)
	Eating behavior	2 (4)
	Physical activity	2 (4)
**Desired frequency of lifestyle follow-up^a^**		
	Daily	0 (0)
	Once a week	6 (12)
	Once a month	26 (53)
	On request	14 (29)
	Other	3 (6)
**A smartphone app might support a healthy lifestyle**		
	Yes	45 (90)
	No	5 (10)
**Preferred content of a smartphone app supporting lifestyle**		
	Eating behavior	46 (92)
	Physical activity	28 (56)
	Weight	38 (76)
	Mental wellbeing	16 (32)
**Preferred mHealth tools to support healthy eating**		
	Eating diary	35 (70)
	Weekly shopping list	37 (74)
	Pictures of portion sizes	31 (62)
	List of allowed foods in pregnancy	50 (100)
**Preferred frequency to complete an eating diary^a^**		
	Never	6 (12)
	A few times a month	4 (8)
	Once a week	3 (6)
	A few times a week	10 (20)
	Every day	26 (53)
**Preferred method of self-monitoring of physical activity**		
	Pedometer	35 (70)
	Registration of physical activity duration in a smartphone app	37 (74)
**Preferred follow-up of mental wellbeing**		
	Registration on a Likert scale with emoticons	45 (90)
	Receiving motivating messages	39 (78)
**Preferred display of weight in the app**		
	Weight + weight gain	36 (72)
	Only weight	11 (22)
	Only weight gain	3 (6)
**Preferred frequency of self-weighing**		
	Never	2 (4)
	Once a week	32 (64)
	Twice a week	4 (8)
	3-6 times a week	6 (12)
	Daily	6 (12)

^a^One survey was missing.

## Discussion

### Principal Findings

This paper reports on the development and evaluation of a mHealth app designed to help women improve their lifestyle during and between pregnancies. We found that pregnant women and health care providers valued the combination of the INTER-ACT app with face-to-face contact in supporting a healthy lifestyle. Personalized feedback from the system with different frequencies according to the focus of health behavior is highly appreciated and increases awareness about healthy behavior. Health care providers stress the importance of considering the vulnerability of risk groups within their cultural and religious contexts when introducing mHealth apps. On one hand, midwives were keen to improve knowledge and skills about sensitive communication and were interested in tools to enhance the intrinsic motivation for behavioral change. On the other hand, they reported reluctance to integrate new technologies fearing a high practical and administrative workload.

### Comparison With Prior Work

Few studies have been published about app development processes for weight management in pregnant women [22,23]. Some studies focused on preconception health only [24,25]; however, to the best of our knowledge, there are no studies on app development targeting women in the interpregnancy period.

Participants in this study reported the need for mHealth as an addition to face-to face contact. This is supported by the findings in a recent RCT comparing the effectiveness of face-to-face contact, that of mHealth, and that of a combination of face-to-face contact with mHealth for 5% weight loss in an obese population. They concluded that a conventional face-to-face weight loss program can partially be replenished with an mHealth program without losing effectiveness [26].

A healthy prepregnancy BMI is an important indicator for optimal pregnancy and birth outcomes [27]. Reaching women with unhealthy lifestyles in due time is a challenge. The effects of preconception interventions for improving pregnancy outcomes in overweight and obese women are scarce [28]. Concurrently, health care providers indicate that they need more training and education about effective obesity communication and weight management practice [29,30]. Women themselves felt that tailored advice specific to their personal situation and weight monitoring would help them implement changes [31]. Both conclusions have been confirmed in this study.

Hence, we developed the INTER-ACT protocol consisting of a mHealth-supported lifestyle program [19]. The INTER-ACT app monitors women’s weight and physical activity through connections with a weighing scale and activity tracker. Eating behavior and mental wellbeing were both self-reported. According to the data, algorithms provide continuous coaching through positive behavioral change techniques. The app targets women with excessive weight gain in a previous pregnancy and can be a low-cost alternative to labor-intensive face-to-face programs for the prevention of postnatal weight retention and excessive gestational weight gain in the subsequent pregnancy. Well-designed intervention trials with attention to structure, method of information delivery, and look and feel are required to further assess the feasibility and effects of such a technology for this target population.

A recent pilot mHealth-supported intervention study that included 40 postnatal women (6-16 weeks) showed that a higher intervention adherence was associated with greatly lower body weight and percentage body fat [32]. It is known that self-monitoring and increased intervention adherence are associated with increased weight loss [33,34]. Concurrently, Herring and colleagues [35] showed that peer support and interaction by social networking in the mHealth app can increase intervention adherence in urban low-income mothers. The high variability in intervention adherence in both mHealth- [32] and non–mHealth-supported lifestyle interventions [7] indicates that it is important to work on these barriers in the future through cocreation with end users.

### Strengths and Limitations

A strength of this study is the mixed methods design used to explore the experiences and views of different health care providers, as well as pregnant women and mothers in the postnatal period. The iterative approach with user participation allowed us to adapt the content and functionality of the app. Limitations are possible biases for the results because of the selection of experienced health care providers and motivated women in the pilot study. Besides, a rather short timeframe for the field evaluation of 3 weeks complicated the technical readiness of the app and thus could influence the crucial adherence and compliance of the program in the longer run. Furthermore, developing tailored feedback is complex and needs more time than was used in this approach to reach deeper levels. However, actual user evaluation showed that the INTER-ACT app increased the awareness for behavioral change.

Recommendations for upgrading the app include subsequent iterations with focus on graphical design, improving stability and performance, making notifications and reminders configurable, and achieving optimal adherence and compliance for using the app and coaching program. Furthermore, an RCT is needed to validate the app, including the coaching program, for long-term use and health-related outcomes.

### Conclusion

Health care providers appreciate the INTER-ACT app in combination with face-to-face contact and emphasize the importance of paying attention to reach the most vulnerable groups, and they are keen on enhancing their sensitive communication skills. On the other hand, they are reluctant to take up additional administrative tasks and to handle technical issues that might be accompanied with the implementation of the INTER-ACT app.

Pregnant women and postnatal mothers value the combination of the INTER-ACT app with face-to-face coaching over more commercial and visually attractive apps. Technological readiness is crucial to refine the app before integration in an RCT. Future studies should evaluate the effectiveness of combinations of face-to-face programs and mHealth apps for this targeted population at risk.

## Abbreviations

BMI: body mass index
mHealth: mobile health
RCT: randomized controlled trial
